# Nystagmus Associated with Carbamazepine Toxicity

**DOI:** 10.5811/cpcem.2017.6.34772

**Published:** 2017-10-18

**Authors:** Lauran Wirfs, Kristen Whitworth, Jesse Kellar

**Affiliations:** * Midwestern University, Chicago College of Osteopathic Medicine, Downers Grove, Illinois; † Lakeland Health, Department of Emergency Medicine, Saint Joseph, Michigan

## CASE PRESENTATION

A 42-year-old male with past medical history significant for epilepsy presented to the emergency department (ED) complaining of dizziness, difficult ambulation, and blurred vision. Vitals were only significant for a blood pressure of 143/89 mm Hg. His neurogenic exam revealed gaze-evoked nystagmus (GEN) (Video) as well as subtle dysmetria and ataxia. The remainder of his physical exam was unremarkable. Computed tomography of the head without contrast showed no acute intracranial abnormality. Upon further questioning, it was discovered he had been unintentionally doubling his dose of carbamazepine 400mg BID because two physicians had prescribed him the medication at the same time. His laboratory studies showed an elevated carbamazepine level at 18.2 ug/mL (normal range 4–12 ug/mL), and he was admitted for observation. The next morning all his symptoms (including his nystagmus) had resolved.

## DIAGNOSIS

### Nystagmus Associated with Carbamazepine Toxicity

Mild GEN may be seen in the normal population with no underlying pathological cause.^1^–^2^ However, exaggerated GEN is pathologic and can be caused by drugs, structural brain abnormalities, or certain diseases (e.g. myasthenia gravis, or demyelinating diseases).^2^–^3^ Carbamazepine, whose structure is related to tricyclic antidepressants, is an anticonvulsant drug used in the treatment of epilepsy, bipolar affective disorders, and trigeminal neuralgia.^4^–^6^ The plasma-level therapeutic range of carbamazepine effective for seizure prophylaxis is 4–12 ug/mL. However, even at higher ends of the therapeutic range, patients can experience adverse effects such as diplopia, blurred vision, nystagmus, or ataxia.^4^ In severe toxicity, carbamazepine may induce seizures or altered level of consciousness, and progress to coma.^4^–^7^ Treatment of carbamazepine toxicity consists of trending serum levels of carbamazepine and supportive care with intravenous fluids as needed for hypotension and benzodiazepines to control seizures.^7^ Patients should be monitored until signs and symptoms of toxicity resolve, and they are deemed medically stable.^5^–^7^

**CPC-EM Capsule**

What do we already know about this clinical entity?

Carbamazepine is an anticonvulsant used to treat epilepsy, bipolar disorder and trigeminal neuralgia. High doses can elicit diplopia, blurred vision, nystagmus, or ataxia. Severe toxicity may induce seizures and progress to coma.

What is the major impact of the image(s)?

This video exhibits exaggerated gaze-evoked nystagmus (GEN) secondary to carbamazepine toxicity.

How might this improve emergency medicine practice?

Mild GEN can occur with no underlying pathology. Exaggerated GEN is pathologic and can be caused by drugs, structural brain abnormalities, or disease. The physician should consider these differentials in patients with exaggerated GEN.

## Supplementary Information

Video Gaze-evoked nystagmus on exam.
